# Exploring creative thinking skills in PISA: an ecological perspective on high-performing countries

**DOI:** 10.3389/fpsyg.2025.1554654

**Published:** 2025-07-09

**Authors:** Sevinc Gelmez Burakgazi, Michael J. Reiss

**Affiliations:** ^1^Department of Educational Sciences, Hacettepe University, Ankara, Türkiye; ^2^Curriculum, Pegagogy and Assessment, Institute of Education, University College London, London, United Kingdom

**Keywords:** creative thinking skills, PISA, international educational assessments, ecological systems theory, Bronfenbrenner, educational policy, creativity awareness

## Abstract

**Introduction:**

Creative thinking is a growing focus in educational reforms worldwide. Extensive research explores its development, measurement, various conceptions, and pedagogical approaches like creative teaching and teaching for creativity. A significant development in this area was the inclusion of creative thinking as an innovation domain in the 2022 Programme for International Student Assessment (PISA). The PISA 2022 assessment sought to evaluate the ability of 15-year-old students across 64 jurisdictions to generate, evaluate, and improve original and diverse ideas.

**Methods:**

This paper examines the key findings of the PISA 2022 creative thinking results from three high-performing jurisdictions in different parts of the world: Singapore, Canada, and Finland. We use Bronfenbrenner’s ecological perspective to understand the interplay of various systemic influences on students’ creative thinking abilities within these educational contexts.

**Results:**

Our analysis, informed by Bronfenbrenner’s framework, highlights how different ecological systems may contribute to their observed outcomes.

**Discussion:**

While acknowledging the complexities and potential pitfalls of directly transferring educational policies between countries, this paper discusses the implications of these findings for school education. We suggest that researchers, policymakers, and educators can gain valuable insights by examining the policies, contexts, and practices of these high-performing nations through an ecological lens, fostering a deeper understanding of how to nurture creative thinking in diverse educational settings.

## 1 Introduction

The world is in a state of continuous innovation. Today’s competitive world requires students who are equipped for the demands of the knowledge-society, sustainability, and changing job markets. From Ken Robinson’s hallmark 2006 TED talk “Do schools kill creativity?” to the present day, creativity is increasingly acknowledged to be one of the core learning competencies required in the 21st century. High-stakes international tests like the Programme for International Student Assessment (PISA) and the Trends in International Mathematics and Science Study (TIMSS), the demands of knowledge societies and a focus on different educational directions [e.g., sustainability, Artificial Intelligence, STEAM (Science, Technology, Engineering, Arts, Mathematics) education, the ongoing space race, etc.] have all led to demands for creativity to be incorporated into school education. An emphasis on students’ creativity has therefore emerged as an increasingly important theme in curriculum development (Kettler et al., 2021; Lim et al., 2014) and has a place in research studies, government reports and policies (Patston et al., 2021).

At the same time, researchers agree that there is substantial confusion and a lack of consensus on the definition of creativity as a construct (Kind and Kind, 2007). There have been a number of attempts formally to define creativity (Beghetto and Kaufman, 2014; Runco, 2004; Runco and Jaeger, 2012; Sawyer, 2003; Sternberg, 2018). For instance, Simonton (2012) proposed a definition based on the three criteria used by the United States Patent Office to evaluate applications for patent protection, while, within education (Sternberg, 2018) defined creativity as: “an attitude toward life and one’s work, but also has cognitive, affective, motivational, and environmental components” (p. 50).

Four types of creativity are often identified: Big-C creativity entails clear-cut, eminent, artistic, and/or revolutionary contributions (like those made by a Picasso or Tesla); Pro-c creativity refers to professional expertise; little-c creativity is the creativity that may be found in everyday life; while mini-c creativity is the novel and personally meaningful understanding of experiences, behavior, and events (Kaufman and Beghetto, 2009). It can be argued that both little-c and mini-c creativity, sometimes named collectively as “small-c creativity” (Gardner, 2011), can be developed in most individuals. Despite all these efforts to arrive at a consensus as to the meaning of creativity, it looks like AI technology may disrupt existing agreements, as advancements in AI technology are impacting creativity research, leading to new methodologies and insights (Wingström et al., 2024).

In schools, little-c (everyday) creativity can be developed through subject-based or cross-curricular approaches. To illustrate subject-based ways to develop everyday creativity, (Rodríguez et al., 2019) maintain that science lessons based on inquiry-based learning help nurture students’ everyday creativity. In terms of cross-curricular ways (Lemons, 2005) suggested that teachers should utilize improvisation techniques to encourage teamwork and risk-taking among students through such approaches as non-competitive games and classroom discussions, so as to cultivate everyday creativity. Additionally, teacher scaffolding through inquiry-based questioning strategies (Hathcock et al., 2015), developing students’ creative metacognition (i.e., self-awareness and self-cognition for monitoring and developing creativity), providing opportunities for imagination, choices and discovery, and modelling creativity (Beghetto and Kaufman, 2014) are some of the ways in which teachers can cultivate the everyday creativity of their students. More generally, Amabile (2018) argued that creativity is influenced by four factors: domain-relevant skills (expertise), creativity-relevant processes (personal approach to a given problem), task motivation (willingness to engage), and the social environment (extra-individual factors).

In her influential study, Kim (2011) argued that there is a “crisis in creativity”, pointing to the need for education to do more to promote student creativity. She analyzed creativity data from age groups of kindergarten through to adults, concluding that there was a decrease in creativity across the age groups in different divergent production abilities like fluency, elaboration, and originality. A subsequent study that she undertook (Kim, 2017) concluded that the creativity crisis is only getting worse. However, Kim’s claim has been criticized as her data used the Torrance Test of Creativity Thinking (TTCT), which some see as problematic, given the cultural context in which this test was developed (Barbot and Said-Metwaly, 2021). After their re-analysis of the data in Kim (2011) and Barbot and Said-Metwaly (2021) concluded that there is no general decline in creativity as individuals get older. More generally, it has been found that research on the development of creativity across the lifespan has produced inconsistent findings (Welter et al., 2017). Rather than a general decline, changes in creativity in different domains have been attributed to a range of factors (Weinstein et al., 2014). For instance, Weinstein et al. (2014) revealed differences between visual artworks and creative writing regarding changes in levels of creativity. Similarly, Lucas et al. (2013) discovered significant differences in creativity depending on the grade level of students.

Whatever the precise factors that affect creativity, the good news is that it is widely accepted that creativity can be taught (Guilford, 1967; Sternberg and Williams, 1996) and developed through school education (Beghetto and Kaufman, 2014; Davis et al., 2013; de Souza Fleith, 2000; Guilford, 1967; Park et al., 2006; Soh, 2015, 2017; Sternberg and Williams, 1996; Sternberg, 2006). Developing students’ creativity has been a longstanding concern (Dewey, 1958; Guilford, 1950; Vygotsky, 2004) and is not straightforward (Barbot et al., 2016; Bijvoet-van Den Berg and Hoicka, 2014; Gralewski et al., 2016; Torrance, 1968; Urban, 1991). Indeed, some teachers have been found to hold negative views about teaching for creativity due to the student personality traits that some teachers believe are associated with creativity (e.g., the student tries to do what others think is impossible, the student is nonconformist, etc.) (Westby and Dawson, 1995). Some teachers hold misconceptions about the nature of creativity and about creative students (Aljughaiman and Mowrer-Reynolds, 2005; Chan and Chan, 1999; Gralewski et al., 2016) or exhibit fixed and unhelpful mindsets concerning creativity (Paek and Sumners, 2019) and are ineffective when teaching for creativity (Kampylis et al., 2009). What is more, there have been few studies on creativity-supportive learning environments (Beghetto and Kaufman, 2014) and there is limited guidance for teachers (Beghetto, 2010), which means that teachers are underprepared and school students still lack effective ways of teaching for creativity (Kaplan, 2019).

The significance of awareness for creativity should not be underestimated. Creativity awareness can be defined as the mindset and predisposition towards creativity, grounded in one’s beliefs and emotions (cf. Karwowski, 2023), and demonstrated through various skills and intentions to act creatively. Understandably, the term ‘creativity awareness’ is normally used in respect of individuals. However, a more systemic understanding, such as that provide by Bronfenbrenner (1977) ecological systems theory, which we discuss below, sees each individual as embedded in a set of concentric discs, each of which exerts its effects on the discs that lie inside it. This means that to enhance school students’ creativity, we need to think about the role of their teachers, of the schools in which teachers teach, and of the national education system within which schools operate, even international zeitgeists that cause enthusiasm for creativity to wax or wane.

In this study, we focus on several key factors, such as the structure of the education system, teacher autonomy, and teacher status, that are closely connected to our research aim of exploring how different national contexts support or limit the development of creativity in schools. These factors were selected because they reflect important aspects of the educational ecology in each country. The education system shapes the overall policy and curriculum priorities; teacher autonomy is important for enabling flexible and creative classroom practices (Lin and Gao, 2023); and teacher status influences motivation, trust, and professional freedom of teachers (Olsen and Mason, 2023). Our choice of these factors was also informed by the previous literature (van der Zanden et al., 2020) and international policy discussions, which often highlight them as essential for promoting creativity in education. By examining these factors, we aim to provide a more comprehensive understanding of how different layers of the education environment—from national policies to classroom realities—interact and influence the creative learning experiences of students.

In this paper we therefore examine a recent major international study (OECD, 2023) on creativity in school students and look at the factors that seem to predict high scores of student creativity. This is not, of course, an intervention study but a cross-comparative correlational study. Furthermore, the community lacks validated quantitative measures of most of the likely key factors. Accordingly, much of what follow is qualitative, an approach that has been used in other education studies comparing high-performing jurisdictions (Hollins and Reiss, 2016). A different analytical approach would be to compare countries that score highly with those that score poorly. However, the more countries that are included in a qualitative study, the lower the resolution for each. Moreover, there is a well-established tradition of focusing on high-performing jurisdictions in such international comparative studies (Hollins and Reiss, 2016; Lau and Ho, 2022; Lau and Lam, 2017). Overall, our study is best seen as an exploratory one, one that we hope proves helpful to researchers, policymakers, and educators who wish to understand, first, the factors that affect creativity in school students (creativity here taken as a combination of little-c and mini-c creativity), and, secondly, what might be done to promote creativity in school students.

### 1.1 An ecological model of human development

Bronfenbrenner’s ecological systems theory is a comprehensive framework for understanding human development within the context of various social environments (Bronfenbrenner and Morris, 2007). The theory has an influential framework that explores the complex relationships between an individual and their varying systems within their environment, each having a distinct role. The theory recognizes that individuals are not isolated beings; rather, they are deeply influenced by the interactions and relationships they have with their surrounding ecological systems. Bronfenbrenner arranged them based on their respective levels of influence on a child (Guy-Evans, 2020).

Bronfenbrenner’s ecological systems theory makes use of a number of different but interconnected systems, namely: the microsystem, mesosystem, exosystem, macrosystem and chronosystem. His framework has been widely used in both educational research and studies of human development more generally (Gelmez Burakgazi, 2025). Clearly, for a child, the family environment (microsystem) is an important factor for their development. Parenting styles and family roles vary across cultures; for example, some cultures favor authoritarian parenting, while others prefer authoritative or permissive approaches. These differences in parenting styles can shape children’s learning and other aspects of their development (Darling, 2007).

The mesosystem, which includes the relationships between different microsystems such as home and school, is also influenced by cultural attitudes towards education. Different cultures have different beliefs about the purpose of education, such as the role of teachers, and the balance between academic studies and extracurricular activities. For instance, some cultures have schools that embody behavioral approaches, with teacher-centered environments, and standardized testing, while other cultures have schools that focus on more holistic approaches to teaching, with more student-centered environments.

The exosystem and macrosystem encompass broader societal and cultural influences including societal structures, economic conditions, and social policies, all of which can affect the resources available to schools and families. Finally, the chronosystem considers changes over time, which can be influenced by cultural shifts. As cultures evolve, so do their educational practices and societal expectations. Accordingly, an understanding of historical and cultural contexts is essential for applying the ecological model validly and effectively. For instance, changing attitudes towards gender roles and inclusion can significantly impact educational policies and practices over time. In multicultural or international educational settings, educators must adapt their approaches to accommodate diverse cultural backgrounds. This may entail integrating culturally relevant materials, promoting cultural competence among educators, and fostering an inclusive environment that values diversity.

## 2 Materials and methods

The PISA 2022 creative thinking test featured 32 tasks assessing three ideation processes: generating diverse ideas, generating creative ideas, and evaluating and improving ideas. It measured both divergent and convergent cognitive processes linked to everyday ‘little-c’ creativity in 15-year-old students worldwide. The test included tasks in four domain contexts: written expression, visual expression, social problem solving, and scientific problem solving, reflecting the need for knowledge and practice in specific areas (OECD, 2023). The PISA 2022 Assessment and Analytical Framework presents definitions and more detailed descriptions of the subjects assessed in PISA (OECD, 2023), subsumed within the overall philosophy that: “Creative thinking is defined as students’ ability to engage productively in the generation, evaluation and improvement of ideas that can result in original and effective solutions, advances in knowledge and impactful expressions of imagination.” (p. 40).

In addition to data from the Creative Thinking test, PISA 2022 collected self-reported data from students, teachers, and school principals via questionnaires. In PISA 2022, these questionnaires gathered data on various possible enablers and drivers of creative thinking, that were not directly measured by the test itself (OECD, 2023). Data were gathered on: students’ curiosity and exploration; students’ creative self-efficacy; students’ beliefs about creativity; creative activities in the classroom and school; and the social environment (student-teacher interactions and the overall school environment) (OECD, 2023).

**Figure 1:**
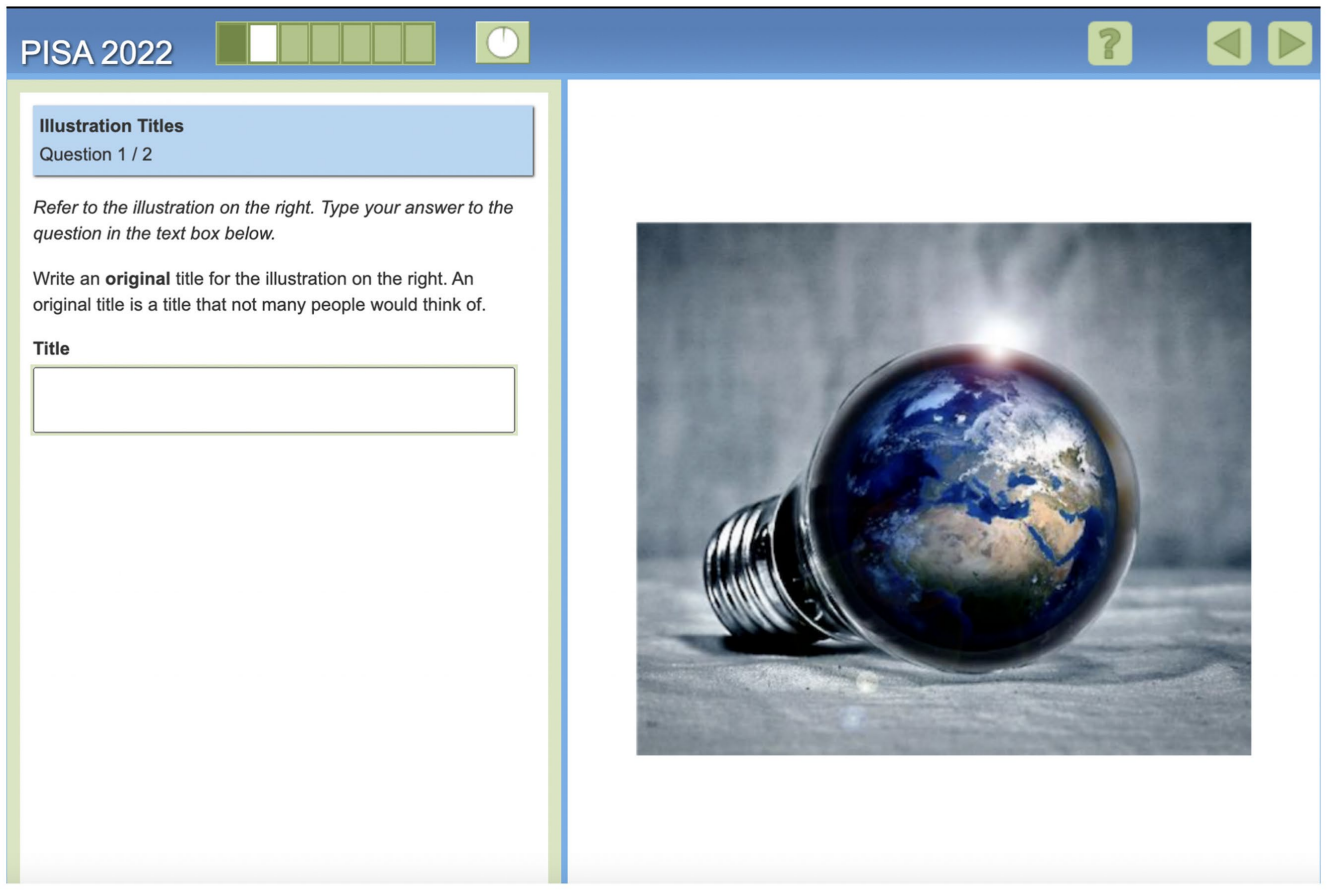
A sample question from the PISA 2022 creative thinking test. Source: https://pisa2022-questions.oecd.org/platform/index.html?user=&domain=CRT&unit=T300-IllustrationTitles&lang=eng-ZZZ.

The 2022 Creative Thinking test was conducted using computer-based assessments, totaling 2 h per student. Generally, in each jurisdiction,^1^ 94% of students were given tests that included 60 min focused on mathematics as the primary domain, along with an additional 60 min dedicated to one of the three secondary or innovative domains, which could be reading, science, or creative thinking. An example of a sample science question is provided in Figure 1.

The 2022 PISA test, initially scheduled for 2021, was postponed by a year due to the COVID-19 pandemic. The extraordinary conditions during this time, which included lockdowns and associated school closures in numerous countries, sometimes made data collection challenging. Although most participating countries adhered to all of PISA’s technical standards, some did not.

In this study, we looked at three countries with high scores in creative thinking from the PISA 2022 assessment. We examined OECD reports, academic articles, and the national education and other websites of these countries for our analysis.

## 3 Results

Table 1 shows the 10 countries, and their scores, that did best on the 2022 PISA Creative Thinking test. For our subsequent analysis, we selected one of the highest performing countries from each of the three continents represented in Table 1: Asia (Singapore), the Americas (Canada) and Europe (Finland). We used Bronfenbrenner’s ecological systems theory as a lens to present our results. The results first present the context and findings for each country; these are then followed by inter-country comparisons.

**Table 1:** PISA 2022 creative thinking performance: the 10 highest-performing countries.

Rank	Country	Score
1	Singapore	41
2	South Korea	38
3	Canada*	38
4	Australia*	37
5	New Zealand	36
6	Estonia	36
7	Finland	36
8	Denmark*	35
9	Latvia*	35
10	Belgium	35

Student performance in creative thinking is reported on a 60-point scale. A country marked with an asterisk (*) indicates that caution is required when interpreting the score, as one or more PISA sampling standards were not met.

Source: OECD (2024a).

### 3.1 Singapore

Singapore, a multicultural and multireligious nation in Southeast Asia, has a population of approximately 5.9 million. The ethnic breakdown of its residents includes 74% Chinese, 14% Malays, 9% Indians, and 3% from other ethnic groups (Department of Statistics Singapore, 2023). Since gaining independence in the 1960s, the People’s Action Party (PAP) has been the main political party, ensuring a stable macrosystem that has supported long-term educational planning and reforms.

The education system in Singapore is traditionally known for its high performance. The country’s education system has been characterized as being centralized at the national level but decentralized at the school level, a characteristic known as ‘centralized decentralization’ (Chua et al., 2019; Tan and Ng, 2007). Singapore gained its independence from Britain in 1967 and a strong examination-driven and teacher-centered culture exists (Koh et al., 2012) where “parents, students, and teachers are all drawn into the competition” (Mee, 1998, p. 196). In recent years, the country has shifted its focus towards fostering creativity and innovation. Students in Singapore achieved a mean score of 41 out of 60 which is significantly higher than the OECD average of 33 in creative thinking (OECD, 2024b). This achievement may be related to certain ongoing reforms in school education in the country. This cultural value of academic excellence represents an influence of the exosystem and macrosystem in shaping educational attitudes and expectations.

One initiative, “holistic student development”, began in 1997 (Datnow et al., 2022). In the contexts of reform movements, the “Teach Less, Learn More” (TLLM) policy was introduced by Singapore’s Ministry of Education in 2004 with the goal of moving from memorization-based learning and high-stakes examinations towards promoting deeper comprehension and analytical thinking skills, as well as fostering creativity. TLLM encourages teachers to apply more student-centered and inquiry-based approaches, facilitating active learning and curiosity. This initiative promotes holistic development and reshapes the classroom microsystem, seeking to produce academically proficient but also problem-solving students in a rapidly changing world.

Another initiative, the “Applied Learning Programme” (ALP) in Singapore aims to bridge the gap between theoretical knowledge and real-world applications in secondary schools (Ministry of Education Singapore, moe.gov.sg). The ALP focuses on experiential and hands-on learning, critical thinking, creativity, and problem-solving skills. By engaging in projects related to fields such as science, technology, engineering, mathematics (STEM), and the arts, the ALP aims to prepare students for future careers by fostering skills that are considered essential in the 21st-century (Ministry of Education Singapore, moe.gov.sg). This initiative strengthens the mesosystem, as it encourages collaboration among teachers, students, and community professionals.

The Learning for Life Programme (LLP) in Singapore complements the ALP by focusing on students’ social–emotional competencies. Through activities such as community service, outdoor education, and leadership training, the LLP helps students cultivate values and skills essential for personal growth and societal contribution. By providing opportunities for holistic development, the LLP is intended to prepare students to navigate and contribute positively to an increasingly complex world. These programs represent important layers of the microsystem and mesosystem, as they connect school experiences with community and personal growth.

Moreover, Singapore’s education system emphasizes stakeholder engagement at multiple ecological levels. These stakeholders include participants at the school level (educators and school leaders); the community level (parents, students, community members, unions, NGOs, media and news agents, community bloggers); the government level (parliament members, representatives of other ministries, government agencies); and the private sector (employers, industry associations, training providers) (Al-Thani, 2024). These layers form part of the exosystem, influencing educational practices indirectly through support structures and expectations.

An example of community-school partnership is COMPASS (Community and Parents in Support of Schools), which was formed in 1998 to strengthen school-parents-community collaboration (Ministry of Education Singapore, 2024). However, although advisory groups are available for input, the influence of individuals at the microsystem level (e.g., teachers, parents, students) on actual decision-making remains limited, showing that while participatory mechanisms exist, power dynamics are still centralized (Deng and Gopinathan, 2016).

In conclusion, Singapore’s high performance in creative thinking can be better understood through Bronfenbrenner’s ecological systems theory, where policies and practices interact across micro-, meso-, exo-, and macro-levels. The country’s education reforms are not isolated actions but are embedded in a broader ecosystem of political stability, cultural expectations, and coordinated stakeholder efforts.

### 3.2 Canada

Canada is divided into 10 provinces and three territories and has a population of around 41 million (Government of Canada, n.d.). Canada is known as having the highest rate of immigration compared to any other country (Government of Canada, n.d.) and sees itself as a multicultural nation. From the view of Bronfenbrenner’s ecological systems theory, Canada’s diverse population plays an important role in shaping students’ learning environments at different system levels. At the microsystem level, students’ daily interactions with peers from various cultural backgrounds influence their social and cognitive development.

Canada’s education system is highly decentralized, with provinces and territories responsible for their own educational policies. The provinces in Canada differ significantly in the school education they provide, not only in their policy instruments but also in their classroom practices. Some provinces focus on standardized tests and set curricula, while others prefer flexible assessment arrangements and innovative teaching methods. At the exosystem level, such provincial decisions—although not directly affecting students—nevertheless indirectly impact classroom structure, available learning tools, and teacher training. At the same time, while each province and territory has its own education system, all aim to provide students with a strong foundation in both academic and practical skills (Peterson, 2023). Canadian schools emphasize critical thinking, creativity, and the benefits of multiculturalism, reflecting the country’s diverse population (The Ontario Curriculum Review and Revision Guide, 2024).

In 2012, the Ministry of Education in British Columbia—one of the largest territories—launched Enabling Innovation: Transforming Curriculum and Assessment to create a more flexible curriculum that promotes creativity and innovation among teachers and students (British Columbia Ministry of Education, 2012). The main goal was to remove obstacles that prevented teachers from customizing learning experiences, with the intention of enabling them to better meet the needs of their students and communities, with a focus on 21st-century skills. The curriculum changes emphasize fewer but more significant learning outcomes, allowing teachers to innovate and personalize learning more effectively. From a mesosystem perspective, this reform promotes stronger collaboration between students, teachers, and communities to shape meaningful learning experiences. This new approach supports personalized learning, creative thinking, and collaboration, with a focus on inclusive teaching practices that embrace diversity in the classroom (British Columbia Ministry of Education, 2012; Milford et al., 2022).

Throughout Canada, emphasis is placed on project-based learning in schools, where students engage in hands-on, collaborative projects that encourage creativity and innovative thinking. The integration of technology in classrooms, combined with a focus on student choice and voice, is intended to support creative learning. These classroom-level practices are part of the microsystem, where daily experiences shape learners’ thinking and behavior. Creativity is also seen as one of the seven core learning competencies required for the 21st century (Boudreault et al., 2013). Programs like STEM and maker education further nurture students’ creative skills by allowing them to explore and experiment with real-world problems.

A more recent initiative began in Ontario in 2024, where the Ministry of Education decided to review its curricula every 5 years. Accordingly, each curriculum will undergo evaluation approximately every 5 years. This revision will proceed in consultation with curriculum developers, parents, teachers, and other stakeholders (NCEE—National Center on Education and the Economy, 2024). This collaboration reflects the mesosystem’s influence, where interactions between school and family or community shape the child’s educational experiences.

Teacher education in Canada focuses on preparing teachers to meet diverse classroom needs and produce high standards (D’Intino and Wang, 2021). Programs vary across provinces but typically include a mix of coursework and practical teaching experience in schools. Emphasis is placed on developing teachers’ abilities to foster inclusive, equitable learning environments (Finkelstein et al., 2021; Specht et al., 2024) and incorporate technology effectively. Within the microsystem, student-teacher interactions during their practicum (teaching practice) shape pre-service teachers’ beliefs and skills in real educational contexts. Ongoing professional development is also encouraged to ensure teachers remain current with educational trends and methodologies (McPherson, 2023). For instance, in Ontario the teacher preparation programs are hosted in universities and have been reformed with the intention of enhancing quality by extending their duration from 1 to 2 years and incorporating a rigorous 80-day practicum (The Ontario Curriculum Review and Revision Guide, 2024). Newly certified teachers in Ontario must then complete a year-long induction program, the New Teacher Induction Program, which provides a reduced teaching load and mentorship from experienced teachers. At the chronosystem level, this reform represents a shift over time in the support structure for early-career teachers. This program supports new teachers with professional development and helps them transition smoothly into their teaching careers. Ontario also emphasizes continuous professional development and has initiatives such as the Ontario Leadership Strategy to foster leadership qualities among educators, ensuring that they can effectively contribute to student achievement and well-being throughout their careers.

In conclusion, Canada’s strong performance in creative thinking can be understood by looking at how different ecological levels support student learning. The decentralized education system (exosystem), multicultural classroom environments (microsystem), and strong partnerships between schools and communities (mesosystem) all contribute to creativity.

### 3.3 Finland

Finland’s decentralized education system is renowned for its emphasis on holistic development and student well-being, which inherently fosters a creative learning environment (Tirri, 2011). Finnish schools focus on interdisciplinary learning, where subjects are taught in a way that relates to real-life contexts, encouraging students to think creatively and critically. The curriculum is flexible, allowing teachers the autonomy to design lessons that inspire creativity. Play-based learning in early childhood education and the integration of arts, music, and crafts throughout schooling further support the development of creative skills. Using Bronfenbrenner’s ecological systems theory, these practices can be interpreted as interactions between different levels of environment. For example, the focus on well-being and creativity in the classroom reflects the microsystem, while teacher autonomy and flexible curriculum policies represent decisions made at the exosystem and macrosystem levels.

The latest curriculum reform in Finland took place between 2012 and 2016. The new national core curriculum was approved in 2014, with local curricula developed and implemented in 2016. Local curricula complemented the national core curriculum and related stakeholders were involved to the process (Finish National Board of Education, 2016). The links between school and community reflect the mesosystem. Key changes in this reform included seven transversal [linking] competences, multidisciplinary learning, diverse learning environments, enhanced student participation and focus on lifelong learning (Vitikka, 2016).

The transversal competences are: thinking and learning to learn; cultural competence, interaction and expression; taking care of oneself and managing daily life; multiliteracy; ICT competence; working life competence and entrepreneurship; and participation and influence, building a sustainable future. The new curriculum also emphasizes a phenomenon-based approach which focuses on real-world issues and problems (Niemi, 2021). Open and flexible school systems are also applied in Finland. This viewpoint supports the idea of ‘learning everywhere’ and the joy of learning. This emphasis on learning across different environments connects to the exosystem and even the chronosystem, where long-term changes in learning culture influence how students experience education across time.

In Finland, becoming a teacher is seen as a prestigious career. The teacher education institutions choose the most appropriate candidates for the profession, which means that typically fewer than 20% of people who apply to be admitted to initial teacher education are successful (Bowles et al., 2014). Teachers need to have a Master’s degree to be a teacher. This high-quality training is part of the microsystem, influencing the direct environment of students, while also shaped by macrosystem values about the importance of education. During their training, future teachers receive extensive pedagogical training, not just to teach classes but to understand the needs of each student. Practical experience and research skills are a big part of the training. Students spend a lot of time in real classrooms, working with experienced teachers. Teachers are trained to use and conduct research to improve their teaching practices. Puustinen et al. (2018) maintain that the concept of the “teacher as a researcher” is taken seriously, while describing critical points in teacher education. They state that even after becoming teachers, Finnish teachers continue to be expected to learn. They participate in regular professional development to keep their skills up-to-date and learn new teaching strategies. For instance, The Council for Creative Education in Finland aims to transform education through creativity, leveraging research and expertise from Finnish academia. It offers customized teacher training and school development programs to improve educational quality, focusing on curriculum, pedagogy, and leadership.

These initiatives show how Finland’s education system is supported at multiple ecological levels—from national education policies (macrosystem) to professional teacher learning networks (mesosystem) and school environments (microsystem). This layered structure creates strong support for creativity in student learning.

### 3.4 Inter-country comparisons

What overall therefore might be the reasons that account for the high performance of 15-year-olds in Singapore, Finland and Canada on PISA’s test of creativity? Table 2 provides a comparison between Singapore, Canada and Finland in terms of the key factors that affect the school education they provide.

**Table 2:** Comparison of key factors in the school education provided in Singapore, Canada and Finland.

Key factors	Singapore	Canada	Finland
Government	Politically stable democracy One-party dominance	Politically stable democracy	Politically stable democracy
Education system	Centralized-decentralized	Decentralized	Decentralized
Last major curriculum revision	2021	Depends on the province/territory	2016
Education policies and approaches	Holistic approach “Teach less, learn more”	Multiculturalism, Holistic learning	Holistic approach Phenomenon-based learning
Teacher autonomy	Low level	High level	High level
Teacher status	High	Quite high	High
Teacher education	Very selective	Quite selective	Very selective
Teacher role in policy	Limited	Actively involved	Actively involved
Parent and student role in school practices	Limited	Actively involved	Actively involved

Sources: Many, with interpretation and synthesis by the authors.

**Figure 2:**
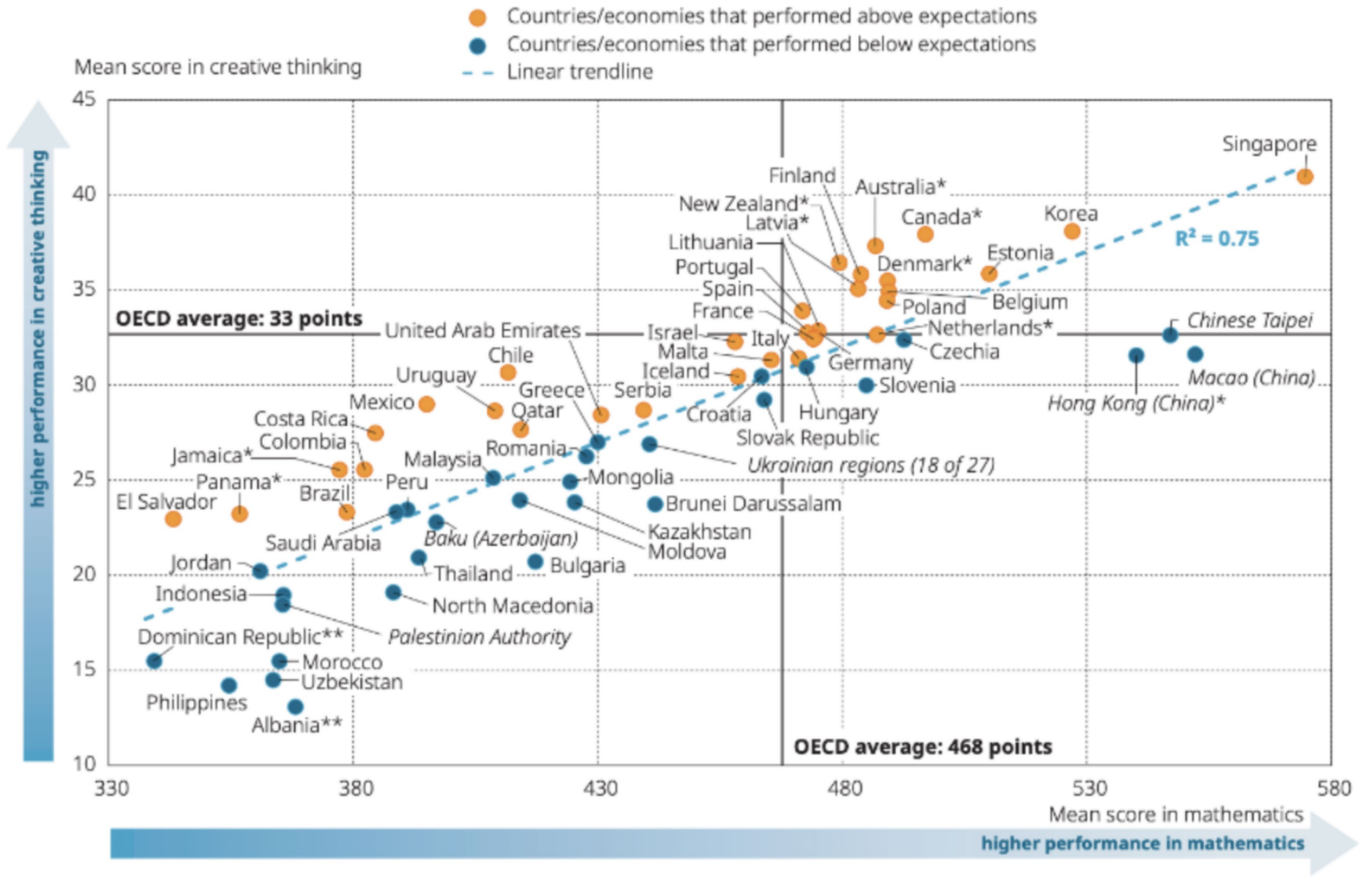
The relationship in the PISA 2022 creativity tests between each jurisdiction’s creativity score and its mathematics score. Source: OECD (2024a).

It is important to emphasize that, as shown in Figure 2, there is a tight correlation across the jurisdictions participating in PISA 2022 between the average academic proficiency of students in a jurisdiction and the average score for creative thinking in that jurisdiction. Nevertheless, the value of R^2^ = 0.75 in Figure 2 means that 25% of the variance in jurisdictions’ scores for creative thinking cannot be explained by the variance in jurisdictions’ scores for academic proficiency in mathematics. Furthermore, within jurisdictions, there will be a range of factors that contribute to creativity beyond academic performance.

Figure 2 suggests that when it comes to Singapore, its high creativity performance may result from the same circumstances and factors that also account for its high mathematics performance (and it comes ‘top’ in reading and in science too). However, the situation is rather different for Finland and Canada. As inspection of Figure 2 shows, each of these countries lies above the average line relating performance in mathematics and in creativity. By way of contrast, China does very well in mathematics, but its three jurisdictions lie some way below the line in terms of its performance in creativity. Indeed, there is now considerable effort being expended in China on how students might be taught to be more creative (Xu et al., 2024a).

There might be in principle be a number of factors responsible for differences in creative thinking scores between countries. Following Bronfenbrenner (1977) these factors operate at a range of levels, which means that if these factors are to be changed, some would require action at government level, whereas, at the other extreme, some can be changed by a single teacher in their classroom. When Singapore, Canada, and Finland are compared (Table 2 and other text above), different educational strengths and challenges that influence their students’ creative thinking abilities are seen, as highlighted in the PISA 2022 creative thinking scores.

In Singapore, the education system is known for its high academic standards, focusing heavily on rote learning and standardized tests. This rigorous approach helps students achieve excellent academic results but might limit their creative thinking due to less emphasis on open-ended problem-solving, inquiry-based learning and other creative tasks. More positively, gender disparities in education are minimal, with both boys and girls performing equally well in academic and creative tasks. The competitive environment ensures that all students have access to high-quality education with a strong focus on discipline and the importance of academic achievement. While this results in academic excellence, it might hinder even greater creativity due to the pressure on students to perform well in formal examinations.

In contrast, Canada takes a more balanced approach, combining academic excellence with fostering creativity. The Canadian education system, despite its diversity, encourages critical thinking and problem-solving, which contributes to higher creative thinking scores. Inclusivity and support for diverse learning needs are emphasized, helping to minimize gender differences and socio-economic disparities. This latter feature is important as students from higher socio-economic status backgrounds (in any country) typically perform better in creative thinking (Acar et al., 2023) due to more extracurricular opportunities and resources. Canadian school students are encouraged to be independent thinkers and problem solvers. Creativity is highly valued and integrated into the school curriculum, reflecting a national attitude that supports innovation and creative expression. Canadian schools provide a supportive environment that encourages experimentation and creative expression. The balance between academic demands and creative freedom helps students perform well in creative thinking tasks.

Finland is renowned for its holistic and student-centered education system, which prioritizes student well-being and minimizes standardized testing. Finnish students enjoy a relaxed and supportive school environment that encourages creativity and independent thinking, all factors that might contribute to high creative thinking scores. The egalitarian approach in the country ensures near equal opportunities for all students, regardless of their socio-economic background, and fosters a positive attitude towards learning and innovation. Students are encouraged to be curious, explore their interests, and engage in innovative thinking from an early age.

## 4 Discussion

The main aim of this study was to explore how different education systems support the development of students’ creativity by focusing on key factors such as teacher autonomy, the role of the curriculum, and the professional status of teachers. By comparing three countries, Finland, Canada, and Singapore, all of which scored highly on PISA 2022 creative thinking test, the study tried to understand how these key factors are shaped by national policies and cultural values, and how they influence teachers’ ability to promote creative thinking in schools. Using Bronfenbrenner’s ecological systems theory, the study looked at creativity as a result of interactions across different levels, such as classroom practices (microsystem), school and community (mesosystem), and education policies and societal expectations (exosystem and macrosystem). This cross-country comparison aimed to provide a deeper understanding of how creativity is supported or limited in different educational contexts.

In today’s world, where computers handle many traditional tasks, humans need to think more creatively and develop innovative solutions. In psychology it is supposed that “all behavior occurs within a context that has the potential to affect it” (Clitheroe et al., 1998, p. 103). Putting the “teacher effect on students’ creativity” to the front, decades of literature on teacher competencies indicate that training teachers in creative methods can lead to more positive attitudes and behaviors toward student creativity (e.g., Houtz and Frankel, 1992; Treffinger, 1995).

Creativity awareness is essential for developing innovative solutions and adapting to new challenges. It involves both recognizing the value of creativity and promoting a creative environment. Such awareness in students, parents, and teachers might help in fostering an educational culture where creativity is not an optional skill but a critical one for real-world problems. Creativity is not, of course, the only thing we want students to gain from their school education. What the precise aims of school education should be are somewhat contentious (e.g., Reiss, 2017) but most educators would include within them the provision of curricula, pedagogies and assessment systems that enable students, by the time they have completed their schooling, to have: a good understanding of a number of subjects, including their first language, mathematics and science, as well as various other subjects (e.g., geography, history, the Arts); an enthusiasm to continue to learn; moral awareness and associated dispositions and behaviors that enable them to value themselves, other individuals, other species and the wider environment; and sufficient preparation to enter the world of paid employment (cf. Heilbronn et al., 2019; Marples and Marples, 1999; Reiss and White, 2013). Creativity can be seen as being likely to contribute to a number of these higher-order aims, though at present this assertion must be presented as a hypothesis rather than a well-established conclusion. For a start, a creative student is more likely to appreciate original thinking in most subjects; indeed, in some subjects, elements of creativity are explicitly required (think designing an open-ended inquiry in science, so-called “creative writing” in one’s first language, musical composition, and so on). Then there is the reality that many employers value creativity (e.g., Clarke, 2023)—which is clearly likely to be related to innovation in a knowledge economy.

Most curricula in OECD countries include in one form or another critical thinking and creativity as expected learning outcomes (Vincent-Lancrin et al., 2019). “Creating New Value” has become one of the three key competencies in the OECD Education 2030 project (OECD, 2018). Comprehensive analysis on curricula in 27 European Union countries found that the term “creativity” is often mentioned (Cachia et al., 2010; Heilmann and Korte, 2010). Cachia et al. (2010) looked specifically at how creativity and innovation are included in curricula across Europe. They found that while some countries have practices that encourage creativity, there is still need for improvement in five key areas: curricula; teaching methods and assessment; teacher training; use of information and communications technology; and the overall educational culture and leadership. A recent study (Patston et al., 2021) analyzed the school curricula in 12 countries, including Finland, and explored the definition of creativity, its place in the curriculum, and concrete advice provided for teachers. The results indicated that even though there is a lot of interest and research in the field of creativity, teachers aren’t getting much support to apply these ideas in their classrooms.

Precisely how creativity might be developed among students no doubt has commonalities across subjects, though subject-specific differences seem likely to exist. For example, in science, it has been argued that “Scientific creativity is a multidimensional construct (Agnoli et al., 2016), encompassing various components, such as scientific knowledge (Yang et al., 2016), motivation in scientific creativity (Taylor and Kaufman, 2021), personality traits that contribute to creativity (Li et al., 2024), divergent thinking (Sak and Ayas, 2013), and convergent thinking (Cropley, 2006; Xu et al., 2024b). Accordingly, Xu et al. (2024b) devised and implemented an inquiry-based intervention in three Chinese secondary school science classrooms over a period of 2 months. Compared to the control group, the intervention group showed statistically significant enhancements in their creativity, specifically in their divergent thinking and convergent thinking. However, it would be premature to conclude that inquiry-based approaches should be used to enhance science creativity. A great deal more work is needed, with a diversity of interventions evaluated so as to establish effect sizes, transferability (what may work in one country or with one age group may not work elsewhere) and unintended consequences (for example, inquiry-based teaching can make high demands on teachers; it would be valuable to establish if there are more straightforward ways of promoting scientific creativity in school).

The findings of Xu et al.’s (2024b) study align with the perspective of Patston et al. (2021) that teachers need more than just being told to “be more creative.” They require clear guidance, practical ideas, and the necessary resources, as highlighted by Kaufman et al. (2022). Furthermore, Beghetto and Kaufman (2014) present a framework for teachers to enhance creativity practices (p. 6): “1. Explicitly teaching for creative thinking; 2. Providing opportunities for choice and discovery; 3. Encouraging students’ intrinsic motivation; 4. Establishing a creativity-supportive learning environment; and 5. Providing opportunities for students to use their imagination while learning.” By fostering creativity awareness through such a framework, teachers can actively engage students in creative processes (Can and Gelmez Burakgazi, 2022). In order to implement a framework like this, teacher training institutions should equip teachers with necessary skills. Teacher education programs thus need “to engage academic teachers with creativity as a hard-edged professional capacity that can and should be fostered through higher education teaching and assessment” (McWilliam and Dawson, 2007, p. 4).

Teachers, as an agent in the microsystem of Bronfenbrenner’s ecological systems theory, with other immediate environments such as home and school, play a crucial role in the development of students’ creativity. For instance, the quality of teacher-student interactions and parental support for creative activities can significantly impact a child’s creativity. This further emphasizes the importance of nurturing creativity awareness in both teachers and parents to create a strong collaboration for fostering student creativity. The mesosystem, which involves the interconnections between different microsystems, highlights the importance of collaboration between parents and teachers to foster creativity. The exosystem, comprising external environments like parents’ workplaces and community resources, influences the support teachers receive to implement creative curricula. National policies and cultural values, encompassed in the macrosystem, also affect creativity development. Countries that prioritize creativity in education, like Singapore, Canada and Finland, show higher performance in PISA creative scores. Lastly, the chronosystem, reflecting changes over time, shows how evolving educational policies impact students’ creative development. This provides encouragement for countries whose students are less creative than desired. In summary, creativity awareness, integrated systematically, can enhance the development of creativity among students.

Overall, this paper achieves a deeper understanding of how creativity is supported in different school educational systems by comparing three countries: Finland, Canada, and Singapore. It shows how key factors, such as teacher autonomy, curriculum flexibility, teacher status, and policy environment, play an important role in supporting or limiting creativity in school settings. The use of Bronfenbrenner’s ecological systems theory helped to examine these factors across different levels, from classroom to national policy, and showed how they interact together. This paper is important because creativity is a key skill in the 21st century, and understanding how different countries successfully support it can help improve education practices more widely. The paper also addresses the need for a holistic view when thinking about creativity in education, not only focusing on the teacher or student but looking at the full educational ecosystem.

## Data Availability

Publicly available datasets were analyzed in this study. This data can be found here: PISA 2022 documents.

## Footnotes

^1^PISA uses the term “jurisdiction” as not all participating units are countries. For example, Hong Kong and Macao are recognised as jurisdictions, as is mainland China.

## References

[ref1] Acar S. Tadik H. Uysal R. Myers D. Inetas B. (2023). Socio-economic status and creativity: a meta-analysis. J. Creat. Behav. 57, 138–172. doi: 10.1002/jocb.568

[ref2] Agnoli S. Corazza G. E. Runco M. A. (2016). Estimating creativity with a multiple-measurement approach within scientific and artistic domains. Creat. Res. J. 28, 171–176. doi: 10.1080/10400419.2016.1162475

[ref3] Aljughaiman A. Mowrer-Reynolds E. (2005). Teachers' conceptions of creativity and creative students. J. Creat. Behav. 39, 17–34. doi: 10.1002/j.2162-6057.2005.tb01247.x

[ref4] Al-Thani G. (2024). Comparative analysis of stakeholder integration in education policy making: case studies of Singapore and Finland. Societies 14:104. doi: 10.3390/soc14070104

[ref9001] Amabile T. M. (2018). Creativity in context: Update to the social psychology of creativity. Routledge.

[ref5] Barbot B. Lubart T. I. Besançon M. (2016). “Peaks, slumps, and bumps”: individual differences in the development of creativity in children and adolescents. New Dir. Child Adolesc. Dev. 2016, 33–45. doi: 10.1002/cad.20152, PMID: 26994723

[ref6] Barbot B. Said-Metwaly S. (2021). Is there really a creativity crisis? A critical review and meta-analytic re-appraisal. J. Creat. Behav. 55, 696–709. doi: 10.1002/jocb.483

[ref7] Beghetto R. A. (2010). “Creativity in the classroom” in The Cambridge handbook of creativity. eds. Kaufman J. C. Sternberg R. J. (Cambridge: Cambridge University Press), 447–464.

[ref8] Beghetto R. A. Kaufman J. C. (2014). Classroom contexts for creativity. High Abil. Stud. 25, 53–69. doi: 10.1080/13598139.2014.905247

[ref9] Bijvoet-van Den Berg S. Hoicka E. (2014). Individual differences and age-related changes in divergent thinking in toddlers and preschoolers. Dev. Psychol. 50, 1629–1639. doi: 10.1037/a0036131, PMID: 24588519

[ref10] Boudreault F.-A. Haga J. Paylor B. Sabourin A. Thomas S. van der Linden C. (2013). Future tense: Adapting Canadian education systems for the 21st century. *An Action Canada Task Force Report*.

[ref11] Bowles T. Hattie J. Dinham S. Scull J. Clinton J. (2014). Proposing a comprehensive model for identifying teaching candidates [article]. Aust. Educ. Res. 41, 365–380. doi: 10.1007/s13384-014-0146-z

[ref12] British Columbia Ministry of Education. (2012). *Enabling Innovation: Transforming curriculum and assessment*. Available online at: https://www.bced.gov.bc.ca/irp/docs/ca%5Ftransformation.pdf (Accessed June 17, 2025).

[ref13] Bronfenbrenner U. (1977). Toward an experimental ecology of human development. Am. Psychol. 32, 513–531. doi: 10.1037/0003-066X.32.7.513

[ref14] Bronfenbrenner U. Morris P. A. (2007). The bioecological model of human development. In Handbook of child psychology: Theoretical models of human development. (6th ed.,) eds. Damon W. Lerner R. M. John Wiley & Sons, 993–1028.

[ref15] Cachia R. Ferrari A. Ala-Mutka K. Punie Y. (2010). *Creative learning and innovative teaching: Final report on the study on creativity and innovation in education in EU member states*.

[ref16] Can İ. Gelmez Burakgazi S. (2022). Training primary school science teachers to be conscious of scientific creativity. Kastamonu Educ. J. 30, 657–668. doi: 10.24106/KEFDERGI-2021-0007

[ref17] Chan D. W. Chan L.-K. (1999). Implicit theories of creativity: teachers' perception of student characteristics in Hong Kong. Creat. Res. J. 12, 185–195. doi: 10.1207/s15326934crj1203_3

[ref18] Chua P. M.-H. Toh Y. He S. Jamaludin A. Hung D. (2019). “Centralised-decentralisation in Singapore education policymaking” in Innovations in educational change: Cultivating ecologies for schools. eds. Hung D. Lee S.-S. Toh Y. Jamaludin A. Wu L. (Singapore: Springer Nature Singapore), 3–21.

[ref19] Clarke E. (2023). “Problems delivering the skills employers want? Creativity-a case in point” in How to enable the employability of university graduates. eds. Hansen S. Daniels K. (Cheltenham: Edward Elgar Publishing), 56–66. doi: 10.4337/9781803926513.00018

[ref20] Clitheroe H. C. Stokols D. Zmuidzinas M. (1998). Conceptualizing the context of environment and behaviour. J. Environ. Psychol. 18, 103–112. doi: 10.1006/jevp.1998.0091

[ref21] Cropley A. (2006). In praise of convergent thinking. Creat. Res. J. 18, 391–404. doi: 10.1207/s15326934crj1803_13

[ref22] D’Intino J. S. Wang L. (2021). Differentiated instruction: a review of teacher education practices for Canadian pre-service elementary school teachers. J. Educ. Teach. 47, 668–681. doi: 10.1080/02607476.2021.1951603

[ref23] Darling N. (2007). Ecological systems theory: the person in the center of the circles. Res. Hum. Dev. 4, 203–217. doi: 10.1080/15427600701663023

[ref24] Datnow A. Park V. Peurach D. J. Spillane J. P. (2022). Transforming education for holistic student development, learning from education system (re) building around the world.

[ref25] Davis G. A. Rimm S. B. Siegle D. B. (2013). Education of the gifted and talented: Pearson new international edition. New York, NY: Pearson Higher Ed.

[ref26] de Souza Fleith D. (2000). Teacher and student perceptions of creativity in the classroom environment. Roeper Rev. 22, 148–153. doi: 10.1080/02783190009554022

[ref27] Deng Z. Gopinathan S. (2016). PISA and high-performing education systems: explaining Singapore’s education success. Comp. Educ. 52, 449–472. doi: 10.1080/03050068.2016.1219535

[ref9003] Department of Statistics Singapore. Yearbook of statistics Singapore. (2023). Available at: https://www.singstat.gov.sg

[ref28] Dewey J. (1958). My pedagogic creed (1897).

[ref29] Finish National Board of Education. (2016). Available online at: https://www.oph.fi/sites/default/files/documents/new-national-core-curriculum-for-basic-education.pdf (Accessed June 17, 2025).

[ref30] Finkelstein S. Sharma U. Furlonger B. (2021). The inclusive practices of classroom teachers: a scoping review and thematic analysis. Int. J. Incl. Educ. 25, 735–762. doi: 10.1080/13603116.2019.1572232

[ref31] Gardner H. (2011). Creating minds: An anatomy of creativity seen through the lives of Freud, Einstein, Picasso, Stravinsky, Eliot, Graham, and Ghandi. New York, NY: Civitas Books.

[ref32] Gelmez Burakgazi S. (2025). “The ecological model of human development” in Exploring adult education through learning theory. ed. Findik L. Y.. (Hershey, PA: IGI Global), 325–342.

[ref33] Government of Canada. (n.d.). Available online at: https://www.canada.ca/en/immigration-refugees-citizenship/corporate/publications-manuals/discover-canada/read-online/canadas-regions.html (Accessed June 17, 2025).

[ref34] Gralewski J. Lebuda I. Gajda A. Jankowska D. M. Wiśniewska E. (2016). Slumps and jumps: another look at developmental changes in creative abilities. Creativity 3, 152–177. doi: 10.1515/ctra-2016-0011

[ref35] Guilford J. (1950). Creativity. Am. Psychol. 5, 444–454. doi: 10.1037/h0063487, PMID: 14771441

[ref36] Guilford J. P. (1967). Creativity: yesterday, today, and tomorrow. J. Creat. Behav. 1, 3–14. doi: 10.1002/j.2162-6057.1967.tb00002.x

[ref37] Guy-Evans O. (2020). Bronfenbrenner's ecological systems theory. *Simply Psychology*.

[ref38] Hathcock S. J. Dickerson D. L. Eckhoff A. Katsioloudis P. (2015). Scaffolding for creative product possibilities in a design-based STEM activity. Res. Sci. Educ. 45, 727–748. doi: 10.1007/s11165-014-9437-7

[ref39] Heilbronn R. Orchard J. Haydon G. (2019). “Aims of education” in Learning to teach in the secondary school (New York, NY: Routledge), 455–466.

[ref40] Heilmann G. Korte W. B. (2010). *The role of creativity and innovation in school curricula in the EU27. A content analysis of curricula documents*.

[ref41] Hollins M. Reiss M. J. (2016). A review of the school science curricula in eleven high achieving jurisdictions. Curric. J. 27, 80–94. doi: 10.1080/09585176.2016.1147968

[ref42] Houtz J. C. Frankel A. D. (1992). Effects of incubation and imagery training on creativity. Creativity Res. J. 5, 183–189. doi: 10.1080/10400419209534432

[ref43] Kampylis P. Berki E. Saariluoma P. (2009). In-service and prospective teachers’ conceptions of creativity. Think. Skills Creat. 4, 15–29. doi: 10.1016/j.tsc.2008.10.001

[ref9002] Kaplan D. E. (2019). Creativity in education: Teaching for creativity development. Psycho. 10, 140–147.

[ref44] Karwowski M. (2023). “Creative mindsets” in The Palgrave encyclopedia of the possible. ed. Glăveanu V. P.. (Cham: Springer), 293–298.

[ref45] Kaufman J. C. Beghetto R. A. (2009). Beyond big and little: the four c model of creativity. Rev. Gen. Psychol. 13, 1–12. doi: 10.1037/a0013688

[ref46] Kaufman J. C. Beghetto R. A. Roberts A. M. (2022). “Creativity in the schools: creativity models and new directions” in Handbook of positive psychology in schools. eds. Allen K.-A. Furlong M. J. Vella-Brodrick D. Suldo S. M. (New York, NY: Routledge), 335–345.

[ref47] Kettler T. Lamb K. N. Mullet D. R. (2021). Developing creativity in the classroom: Learning and innovation for 21st-century schools. New York, NY: Routledge.

[ref48] Kim K. H. (2011). The creativity crisis: the decrease in creative thinking scores on the Torrance tests of creative thinking. Creat. Res. J. 23, 285–295. doi: 10.1080/10400419.2011.627805

[ref49] Kim K. H. (2017). The creativity crisis: it’s getting worse. Available online at: https://www.ideatovalue.com/crea/khkim/2017/04/creativity-crisis-getting-worse/ (Accessed June 17, 2025).

[ref50] Kind P. M. Kind V. (2007). Creativity in science education: Perspectives and challenges for developing school science. Stud. Sci. Edu. 43, 1–37. doi: 10.1080/03057260708560225

[ref51] Koh K. H. Tan C. Ng P. T. (2012). Creating thinking schools through authentic assessment: the case in Singapore. Educ. Assess. Eval. Account. 24, 135–149. doi: 10.1007/s11092-011-9138-y

[ref52] Lau K.-C. Ho S.-C. E. (2022). Attitudes towards science, teaching practices, and science performance in PISA 2015: multilevel analysis of the Chinese and Western top performers. Res. Sci. Educ. 52, 415–426. doi: 10.1007/s11165-020-09954-6

[ref53] Lau K.-C. Lam T. Y.-P. (2017). Instructional practices and science performance of 10 top-performing regions in PISA 2015. Int. J. Sci. Educ. 39, 2128–2149. doi: 10.1080/09500693.2017.1387947

[ref54] Lemons G. (2005). When the horse drinks: enhancing everyday creativity using elements of improvisation. Creat. Res. J. 17, 25–36. doi: 10.1207/s15326934crj1701_3

[ref55] Li J. Zhang J. Bu X. Zhang N. (2024). Does a creative person necessarily exhibit creativity? The interaction between creative personality and positions in social networks. Innovation 26, 188–206. doi: 10.1080/14479338.2022.2105851

[ref56] Lim C. Lee J. Lee S. (2014). A theoretical framework for integrating creativity development into curriculum: the case of a Korean engineering school [article]. Asia Pac. Educ. Rev. 15, 427–442. doi: 10.1007/s12564-014-9334-9

[ref57] Lin Q. Gao X. (2023). Exploring the predictors of teachers’ teaching autonomy: a three-level international study [article]. Teach. Teach. Educ. 135:Article 104338. doi: 10.1016/j.tate.2023.104338

[ref58] Lucas B. Claxton G. Spencer E. (2013). Progression in student creativity in school: First steps towards new forms of formative assessments. Contemp. Read. Law Soc. Justice 81–121.

[ref59] Marples R. Marples R. (1999). The aims of education. London: Routledge.

[ref60] McPherson H. (2023). Reproduction to transformation: disrupting teacher habitus through pedagogic work. Br. J. Sociol. Educ. 44, 355–373. doi: 10.1080/01425692.2022.2161473

[ref61] McWilliam E. Dawson S. (2007). Understanding creativity: A survey of ‘creative’ academic teachers. Canberra, Australia: The Carrick Institute for Learning and Teaching in Higher Education.

[ref62] Mee C. Y. (1998). The examination culture and its impact on literacy innovations: the case of Singapore. Lang. Educ. 12, 192–209. doi: 10.1080/09500789808666748

[ref63] Milford T. Lawrence B. McGhie-Richmond D. Brenton-Haden S. (2022). “Inclusive education in British Columbia: teaching to diversity” in The inclusion for students with special educational needs across the Asia Pacific: The changing landscape. eds. Beamish W. Yuen M. (Singapore: Springer Nature Singapore), 151–168.

[ref9004] Ministry of Education Singapore. Education statistics digest 2024. (2024). Available at: https://www.moe.gov.sg

[ref64] NCEE—National Center on Education and the Economy. (2024). Available online at: https://ncee.org/canada/ (Accessed June 17, 2025).

[ref65] Niemi K. (2021). ‘The best guess for the future?’ Teachers’ adaptation to open and flexible learning environments in Finland. Educ. Inq. 12, 282–300. doi: 10.1080/20004508.2020.1816371

[ref66] OECD. (2018). Transformative Competencies for 2030. Available online at: https://www.oecd.org/content/dam/oecd/en/about/projects/edu/education-2040/concept-notes/Transformative_Competencies_for_2030_concept_note.pdf (Accessed June 17, 2025).

[ref67] OECD. (2023). PISA 2022 Creative Thinking Framework. doi: 10.1787/471ae22e-en

[ref68] OECD. (2024a). PISA 2022 technical report. doi: 10.1787/01820d6d-en

[ref69] OECD. (2024b). PISA Results 2022 (Volume III) – Factsheets: Singapore. Available online at: https://www.oecd.org/en/publications/pisa-results-2022-volume-iii-factsheets_041a90f1-en/singapore_3e8ab415-en.html#:~:text=With%20a%20mean%20score%20of,in%20creative%20thinking%20(33) (Accessed June 17, 2025).

[ref70] Olsen A. A. Mason E. N. (2023). Perceptions of autonomy: differential job satisfaction for general and special educators using a nationally representative dataset [article]. Teach. Teach. Educ. 123:Article 103999. doi: 10.1016/j.tate.2022.103999

[ref71] Paek S. H. Sumners S. E. (2019). The indirect effect of teachers’ creative mindsets on teaching creativity. J. Creat. Behav. 53, 298–311. doi: 10.1002/jocb.180

[ref72] Park S. Lee S.-Y. Oliver J. S. Cramond B. (2006). Changes in Korean science teachers' perceptions of creativity and science teaching after participating in an overseas professional development program. J. Sci. Teach. Educ. 17, 37–64. doi: 10.1007/s10972-006-9009-4

[ref73] Patston T. J. Kaufman J. C. Cropley A. J. Marrone R. (2021). What is creativity in education? A qualitative study of international curricula [article]. J. Adv. Acad. 32, 207–230. doi: 10.1177/1932202X20978356

[ref74] Peterson A. (2023). Education transformation in British Columbia. Case Study. *Center for Universal Education at The Brookings Institution*.

[ref75] Puustinen M. Säntti J. Koski A. Tammi T. (2018). Teaching: a practical or research-based profession? Teacher candidates' approaches to research-based teacher education. Teach. Teach. Educ. 74, 170–179. doi: 10.1016/j.tate.2018.05.004

[ref76] Reiss M. J. (2017). “The curriculum arguments of Michael Young and John White” in Sociology, curriculum studies and professional knowledge (London: Routledge), 121–131.

[ref77] Reiss M. White J. (2013). An aims-based curriculum: The significance of human flourishing for schools. eds. Guile D. Lambert D. Reiss M. J. London: IoE Press.

[ref78] Rodríguez G. Pérez N. Núñez G. Baños J.-E. Carrió M. (2019). Developing creative and research skills through an open and interprofessional inquiry-based learning course. BMC Med. Educ. 19, 1–13. doi: 10.1186/s12909-019-1563-5, PMID: 31068154 PMC6506954

[ref79] Runco M. (2004). “Everyone has creative potential” in Creativity: from potential to realization. eds. Sternberg R. J. Grigorenko E. L. Singer J. L. (American Psychological Association).

[ref80] Runco M. A. Jaeger G. J. (2012). The standard definition of creativity. Creat. Res. J. 24, 92–96. doi: 10.1080/10400419.2012.650092

[ref81] Sak U. Ayas M. B. (2013). Creative scientific ability test (C-SAT): a new measure of scientific creativity. Psychol. Test Assess. Model. 55, 316–329.

[ref82] Sawyer R. K. (2003). “Emergence in creativity and development” in Creativity and development. eds. Sawyer R. K. John-Steiner V. Moran S. Sternberg R. J. Feldman D. H. Nakamura J.. Oxford: Oxford University Press, 12–60.

[ref83] Simonton D. K. (2012). Taking the U.S. patent office criteria seriously: a quantitative three-criterion creativity definition and its implications. Creat. Res. J. 24, 97–106. doi: 10.1080/10400419.2012.676974

[ref84] Soh K. (2015). Creativity fostering teacher behaviour around the world: annotations of studies using the CFTIndex. Cogent Educ. 2:1034494. doi: 10.1080/2331186X.2015.1034494

[ref85] Soh K. (2017). Fostering student creativity through teacher behaviors. Think. Skills Creat. 23, 58–66. doi: 10.1016/j.tsc.2016.11.002

[ref86] Specht J. Fairbrother M. Gallagher T. L. Ismailos L. Villella M. Mac Cormack J. (2024). ‘Learning from what my Mentor teachers were doing in the classroom to include diverse learners’: experiences that contribute to the use of inclusive instruction in pre-service teachers. Int. J. Disabil. Dev. Educ., 1–14. doi: 10.1080/1034912X.2024.2403384

[ref87] Sternberg R. Williams W. M. (1996). How to develop student creativity. Arlington VA: Association for Supervision and Curriculum Development.

[ref88] Sternberg R. J. (2006). The nature of creativity. Creat. Res. J. 18, 87–98. doi: 10.1207/s15326934crj1801_10, PMID: 38329648

[ref89] Sternberg R. J. (2018). A triangular theory of creativity. Psychol. Aesthet. Creat. Arts 12, 50–67. doi: 10.1037/aca0000095

[ref90] Tan C. Ng P. T. (2007). Dynamics of change: decentralised centralism of education in Singapore. J. Educ. Chang. 8, 155–168. doi: 10.1007/s10833-006-9016-4

[ref91] Taylor C. L. Kaufman J. C. (2021). The creative trait motivation scales. Think. Skills Creat. 39:100763. doi: 10.1016/j.tsc.2020.100763

[ref92] The Ontario Curriculum Review and Revision Guide. (2024). Available online at: https://www.dcp.edu.gov.on.ca/en/curriculum-review/process (Accessed June 17, 2025).

[ref93] Tirri K. (2011). Holistic school pedagogy and values: Finnish teachers’ and students’ perspectives. Int. J. Educ. Res. 50, 159–165. doi: 10.1016/j.ijer.2011.07.010, PMID: 40487666

[ref94] Torrance E. P. (1968). A longitudinal examination of the fourth grade slump in creativity. Gift. Child Q. 12, 195–199. doi: 10.1177/001698626801200401

[ref95] Treffinger D. J. (1995). Creative problem solving: overview and educational implications. Educ. Psychol. Rev. 7, 301–312. doi: 10.1007/BF02213375

[ref96] Urban K. K. (1991). Recent trends in creativity research and theory in Western Europe. Eur. J. High Abil. 1, 99–113. doi: 10.1080/0937445900010114

[ref97] van der Zanden P. Meijer P. C. Beghetto R. A. (2020). A review study about creativity in adolescence: where is the social context? Think. Skills Creat. 38:Article 100702. doi: 10.1016/j.tsc.2020.100702, PMID: 40487666

[ref98] Vincent-Lancrin S. González-Sancho C. Bouckaert M. Luca F. D. Fernández-Barrerra M. Jacotin G.. (2019). Fostering students’ creativity and critical thinking. Paris: OECD.

[ref99] Vitikka E. (2016). Available online at: https://finlandabroad.fi/documents/384951/405231/erja_vitikka_redesigning_curriculum_in_finland.pdf/c8a7e4c8-c016-f762-d1d5-66d9e7f00151?t=1551363347184 (Accessed June 17, 2025).

[ref100] Vygotsky L. S. (2004). Imagination and creativity in childhood. J. Russ. East Eur. Psychol. 42, 7–97. doi: 10.1080/10610405.2004.11059210

[ref101] Weinstein E. C. Clark Z. DiBartolomeo D. J. Davis K. (2014). A decline in creativity? It depends on the domain. Creat. Res. J. 26, 174–184. doi: 10.1080/10400419.2014.901082

[ref102] Welter M. M. Jaarsveld S. Lachmann T. (2017). Problem space matters: the development of creativity and intelligence in primary school children. Creat. Res. J. 29, 125–132. doi: 10.1080/10400419.2017.1302769

[ref103] Westby E. L. Dawson V. L. (1995). Creativity: asset or burden in the classroom? Creat. Res. J. 8, 1–10. doi: 10.1207/s15326934crj0801_1

[ref104] Wingström R. Hautala J. Lundman R. (2024). Redefining creativity in the era of AI? Perspectives of computer scientists and new media artists. Creat. Res. J. 36, 177–193. doi: 10.1080/10400419.2022.2107850

[ref105] Xu S. Reiss M. J. Lodge W. (2024a). The development of an analytical model for science classroom creativity in China. Res. Sci. Technol. Educ., 1–26. doi: 10.1080/02635143.2024.2377265

[ref106] Xu S. Reiss M. J. Lodge W. (2024b). Enhancing scientific creativity through an inquiry-based teaching approach in secondary science classrooms. Int. J. Sci. Educ., 1–18. doi: 10.1080/09500693.2024.2419987

[ref107] Yang K.-K. Lee L. Hong Z.-R. Lin H.-S. (2016). Investigation of effective strategies for developing creative science thinking. Int. J. Sci. Educ. 38, 2133–2151. doi: 10.1080/09500693.2016.1230685

